# Advances and Retrogression in Medical Teaching.—Bellevue Hospital Medical College

**Published:** 1881-04

**Authors:** 


### EDITORIAL, ETC.

*Advances and Retrogression in Medical Teaching.—Bellevue Hospital Medical College.*—Bellevue Medical College, New York, lately made an advance movement in education, which it seems was a failure, or at least the Faculty saw fit lately to recede from this position and fall back on the simple qualification, as the only one demanded of students, of a desire to become a physician, and of possession of the necessary fee to pave the way to a realization of this desire. The advance made by Bellevue College was that " the Faculty for the session of 1880–81, adopted the following innovations, namely : 'Matriculation Examination ' The matriculation examination will consist of English composition (one foolscap page of original composition upon any subject in the hand writing of the candidate,) grammar, an examination upon the abovementioned composition; arithmetic, including vulgar and decimal fractions; algebra, including simple equations; geometry, first two books of Euclid. This examination will be waived for those who have received the degree of A. B., those who have passed the freshman examination for entrance into any incorporated literary college; those who present certificates of proficiency in the subjects of the matriculation examination from the principal or teachers of any regular high school; and those who have passed a matriculation examination at any recognized medical college or scientific school or academy in which an examination is required for admission.

The other change was to make a three years' course, instead of two, as formerly, a *sine qua non* for graduation."

The result of this change was that the attendance of students on Bellevue College fell off one-half, and the Faculty, seeing their decimated classes, felt that (we copy from the *Medical Gazette*) " this forces the solution of the question whether a body of medical teachers can, in justice to themselves and their school, afford to continue in operation a policy in which the profession, upon whose recomendations they are dependent for students, do not uphold and support them?

The Bellevue College authorities have decided this question in the negative, and evidently do not propose to offer themselves as a sacrifice on the altar of medical progress."

Evidently these gentlemen have no idea of seeing their school closed for lack of patronage, on account of advanced ideas respecting the previous qualifications of students who may wish to pursue a course of study in such an institution of learning. That such training would amazingly facilitate the labors of a corp of teachers no one can doubt; but if, as they say, those who send students to them do not recognize the need for any advance, it is hardly possible for a college, unless it be an endowed institution, to put a higher standard than is required by the public. In point of fact—and it is what we have always contended for,—the colleges are what the public make them. Get behind the curtain of faculty deliberations, where the graduating of a doubtful man is under discussion, and the question "what will be said by our friends in his neighborhood if we turn out such a man," must and will enter into the discussion. There is no avoiding it. Let the profession at large demand at the hands of the colleges a higher standard of education, under penalty of withdrawal of patronage, and the colleges will not be slow to respond; for "the colleges must be managed on business principles," and this would be no less one of them than is the acceptance of such students as now come, regardless of qualification. What was said by the writer in past years, reflecting the views of a distinguished thinker, is still true, that after all the average doctor of medicine or average dentist is as good as the public taste requires; and the best way to reform these professions is for those upon whom they practice to be well posted as to the qualifications necessary for them.

We copy from a late daily paper the following, which, however erroneous in view, shows the drift of public opinion:

"The medical society of Bangor recently expelled one of its members, a physician in good standing, on the ground that he had been guilty of the professional indiscretion of advertising his business. Had the erring brother been able to wheedle the local editors of Bangor into making complimentary mention of his professional skill, without charge, there would probably have been no trouble; but, having committed the unpardonable offence of having paid for the publication of a business announcement, in which he and the public were alike interested, he had to go. The dignity of the profession could not tolerate an impropriety at once so shocking in itself and so dangerous as a precedent.

The doctors of Bangor, and of the rest of the country as well, ought to know that this sort of thing is unworthy of themselves and of the age in which they live. They can no longer occupy that relation to the rest of the world which their guild sustained to it during medieval times, when learning and general intelligence were far less common than they are now, and which only the 'medicine men' sustain to the other members of the savage tribes of to-day. The time for such assumed superiority and exclusiveness has long since gone by. The world is moving, and the medical profession cannot afford to stand still. There is nothing sacred about it,—nothing that can exempt it from the operation of the same laws which apply to other honorable pursuits in life. The physician of the future must come out of that time-worn and cracked shell of 'professional dignity' upon which his predecessor laid so much stress, and stand the test of publicity and compitition, as is done by architects, lawyers, engineers, and the members of equally learned and honorable professions. If a doctor possesses special skill or knowledge in any particular branch of his profession, it is alike due to him and to the rest of the world that that fact should be publicly known; and if the medical societies forever undertake to obstruct the course of a law of life so natural and so just they will find the confidence and the patronage they have so long enjoyed rapidly and surely passing to other hands.

